# Malaria among migrant populations in the Greater Mekong Subregion: a scoping review of evidence and implications for malaria elimination

**DOI:** 10.1186/s12939-026-02953-5

**Published:** 2026-07-28

**Authors:** Pyae Linn Aung, Kyaw Zay Ya, Ajjima Thussachan, Naing Bo Bo Min, Kimberly Fornace, Liwang Cui, Daniel M. Parker, Jetsumon Sattabongkot

**Affiliations:** 1 Mahidol Vivax Research Unit, Faculty of Tropical Medicine, Mahidol University, Bangkok, Thailand; 2 School of Health and Medical Sciences, City St. George’s University of London, London, UK; 3 Department of Tropical Pediatrics, Faculty of Tropical Medicine, Mahidol University, Bangkok, Thailand; 4 Saw Swee Hock School of Public Health and National University Health System, National University of Singapore, Singapore, Singapore; 5 Division of Infectious Diseases and International Medicine, Department of Internal Medicine, Morsani College of Medicine, University of South Florida, Tampa, FL USA; 6 Department of Global Health & Biobehavioral Sciences, Department of Epidemiology & Biostatistics, Department of Health, Society, & Behavior, University of California, Irvine, CA USA

**Keywords:** Malaria, Migrants, Mobile populations, Greater Mekong Subregion, Healthcare access, Malaria elimination, Scoping review

## Abstract

**Background:**

Countries in the Greater Mekong Subregion (GMS) have made substantial progress toward malaria elimination, with a regional goal of achieving elimination by 2030. However, residual transmission persists, particularly among migrant and mobile populations in border and forested areas, who often experience increased exposure and limited access to healthcare and preventive interventions. This scoping review aimed to synthesize evidence on malaria epidemiology, healthcare access, prevention practices, and health system challenges among migrant and mobile populations in the GMS.

**Methods:**

A scoping review was conducted following PRISMA-ScR guidelines. Two electronic databases (PubMed and Scopus) were searched from inception to March 2026. Studies reporting on malaria among migrant or mobile populations in GMS countries were included. Two reviewers independently screened studies and extracted data. Findings were synthesized narratively and organized into six thematic domains.

**Results:**

A total of 847 records were identified, of which 52 studies met the inclusion criteria. Malaria burden among migrant and mobile populations was generally low but highly heterogeneous, with persistent transmission concentrated in border and forested areas and evidence of asymptomatic and submicroscopic infections indicating hidden reservoirs of transmission. Across the included studies, migrant and mobile populations experienced intersecting structural vulnerabilities, including cross-border mobility, precarious employment, legal and administrative barriers, language barriers, and limited access to health services, that contributed to delayed diagnosis and treatment, inconsistent uptake of preventive measures, and reliance on informal or cross-border healthcare. Preventive practices often remained suboptimal because of occupational and mobility-related constraints. Existing interventions, including community-based services and mobile health approaches, showed promise but remained limited in coverage and evaluation, underscoring persistent challenges for surveillance, service delivery, and cross-border coordination.

**Conclusions:**

Migrant and mobile populations remain a critical challenge to malaria elimination in the GMS. Residual transmission is sustained by asymptomatic infections, structural barriers to healthcare access, suboptimal preventive practices, and high mobility. Achieving malaria elimination by 2030 will require tailored and equitable strategies that strengthen surveillance, expand flexible and accessible service delivery, and enhance cross-border collaboration for migrant and mobile populations.

**Supplementary Information:**

The online version contains supplementary material available at https://doi.org/10.1186/s12939-026-02953-5.

## Introduction

Malaria transmission in the Greater Mekong Subregion (GMS), comprising Cambodia, Lao People’s Democratic Republic, Myanmar, Thailand, and Vietnam, has declined substantially over the past decade due to intensified control efforts, strengthened surveillance systems, and expanded access to diagnosis and treatment [1–4]. According to the World Malaria Report 2025 [5], reported malaria cases in the region decreased from approximately 386,000 in 2014 to 208,000 in 2024. China was certified malaria-free in 2021, representing a major milestone for regional elimination [6]. However, malaria transmission persists in several GMS countries. The majority of cases were reported in Myanmar (189,358 in 2024), followed by Thailand (14,690), while substantially lower case numbers were reported in Cambodia, Lao People’s Democratic Republic, and Vietnam [5]. Notably, recent increases in malaria cases in Myanmar since 2021 have raised concerns about resurgence in parts of the region, with potential implications for cross-border transmission into neighboring countries, including Thailand [7]. Despite regional commitments to eliminate malaria by 2030, residual transmission remains concentrated in border and remote areas where health services and vector control interventions are difficult to sustain [1, 5, 8, 9].

Population mobility and cross-border migration are widely recognized as key challenges to malaria elimination across the Asia-Pacific region, particularly in countries approaching elimination where residual transmission has become increasingly concentrated among mobile and hard-to-reach populations [1, 10, 11]. Within the GMS, large numbers of migrant workers and other mobile populations move across international borders for employment, livelihood, and other reasons, often traveling between areas with differing malaria transmission intensity [11–16]. These movements frequently occur in international border regions characterized by persistent malaria transmission, reflecting the combined influence of ecological conditions, occupational exposures, and differing health system capacities, and challenges in coordinating malaria control across national boundaries. Migrants and migrant populations are at increased risk of malaria because of occupational exposure in endemic environments, temporary or unstable living conditions, and limited access to preventive measures such as insecticide-treated nets (ITNs) [17–25]. In addition, structural and social barriers, including language barriers, legal or documentation status, temporary and highly mobile living and working conditions, and limited familiarity with local health systems, may limit access to malaria prevention, diagnosis, and treatment [1, 11, 13, 18, 26–30]. These intersecting structural vulnerabilities contribute to inequities in access to malaria prevention, diagnosis, and treatment, underscoring the importance of understanding how they collectively influence malaria risk and malaria elimination among migrant and mobile populations in the GMS.

A growing body of research has examined malaria among migrant and mobile populations in the GMS, addressing diverse aspects including epidemiology, healthcare access, and targeted interventions [1, 11]. However, the available evidence remains fragmented across disciplines and is often limited to specific countries, migrant subgroups, or programmatic contexts. As a result, there is a lack of a comprehensive regional synthesis that integrates epidemiological, behavioral, and health system perspectives. This limits a clear understanding of how multiple factors interact to shape malaria risk among migrant populations and constrains the development of coordinated strategies for malaria elimination in the region. Given the critical role of migrant populations in sustaining residual transmission, a comprehensive synthesis of the available evidence is needed. Therefore, this study aimed to conduct a scoping review of malaria among migrant populations in the GMS to synthesize the available evidence and inform policy and programmatic responses. Specifically, the review sought to synthesize evidence on malaria epidemiology and burden, access to healthcare and treatment-seeking, knowledge, perceptions, and behaviors, prevention practices and vector control, mobility- and occupation-related risk factors, and interventions, surveillance, and health system challenges relevant to malaria elimination among migrant populations.

## Methods

### Study design

This scoping review followed established methodological frameworks, including those proposed by Arksey and O’Malley [31] and further refined by the Joanna Briggs Institute [32], and was reported in accordance with the PRISMA-ScR guidelines [33, 34]. No formal review protocol was prospectively registered.

### Eligibility criteria

Studies were considered eligible for inclusion if they met the following criteria. Primary research studies using quantitative, qualitative, or mixed-methods designs published in peer-reviewed journals were eligible for inclusion. First, studies had to focus on malaria and include migrant or mobile populations. For the purpose of this review, migrant populations were defined as individuals who move either across borders or within countries in the GMS for employment, livelihood, or other reasons. This includes cross-border migrant workers, internal migrants, seasonal laborers, and mobile populations engaged in high-risk occupations (e.g., forest-related or agricultural work). Studies involving refugees or displaced populations were included only when ongoing mobility or recent migration was described. For this review, recent migration refers to individuals who had not yet established stable or long-term residence in the destination setting, as indicated by temporary living conditions, recent arrival, or continued movement. Second, studies had to be conducted within countries of the GMS, including Cambodia, Lao People’s Democratic Republic, Myanmar, Thailand, Vietnam, and Yunnan Province of China. Third, studies needed to report evidence related to at least one of the following domains: malaria burden or epidemiology; access to healthcare and treatment-seeking; knowledge, perceptions, and behaviors; prevention practices and vector control; mobility, occupation, and risk factors; or interventions, surveillance, and health system challenges relevant to malaria elimination among migrant populations. These domains were initially developed a priori based on the review objectives and informed by the WHO Global Technical Strategy for Malaria 2016–2030, the WHO Framework for Malaria Elimination, and existing literature on malaria elimination and migrant health in the GMS [35, 36]. During data charting, the domains were further refined iteratively to ensure that they comprehensively captured the range of epidemiological, behavioral, mobility-related, and health system evidence reported across the included studies.

Studies focusing on long-term settled refugee populations (defined as residing in the destination setting for ≥ 12 months without ongoing mobility) were excluded. Studies focusing exclusively on laboratory, molecular, or entomological aspects of malaria without relevance to migrant populations were excluded. Editorials, commentaries, and opinion pieces without primary data were also excluded. No restrictions were applied on the year of publication. Only studies published in English were included. No formal risk of bias assessment was conducted, consistent with scoping review methodology (Table 1).

**Table 1:** Eligibility criteria for study selection

Domain	Inclusion criteria	Exclusion criteria
Study design	Primary quantitative, qualitative, or mixed-methods research	Editorials, commentaries, opinion pieces, conference abstracts, reviews, and studies without primary data
Population	Migrant or mobile populations, including cross-border migrants, internal migrants, seasonal migrants, and other mobile populations in the GMS	Long-term settled refugee populations without ongoing mobility
Setting	Studies conducted in Cambodia, Lao PDR, Myanmar, Thailand, Vietnam, or Yunnan Province of China	Studies conducted outside the GMS
Topic	Malaria and at least one review domain (epidemiology, healthcare access, prevention, mobility/risk factors, interventions, surveillance, or health systems)	Laboratory, molecular, or entomological studies without relevance to migrant populations
Language	English	Non-English

### Information sources and search strategy

A comprehensive literature search was conducted using PubMed and Scopus, two complementary databases that provide broad coverage of biomedical, public health, and multidisciplinary literature. PubMed was selected because of its extensive indexing of biomedical and clinical research, whereas Scopus was included to capture additional multidisciplinary and regional publications not always indexed in PubMed. In addition, the reference lists of all included studies were manually screened to identify any additional eligible publications. Together, these search approaches were considered sufficient to identify the peer-reviewed literature relevant to the objectives of this scoping review.

The search strategy combined keywords and MeSH terms related to malaria, migration or mobile populations, and countries within the GMS. Search terms included combinations of terms such as “malaria,” “*Plasmodium*,” “migrant,” “migration,” “migrant workers,” “mobile populations,” and “cross-border,” together with geographic terms representing countries in the region, including Cambodia, Lao People’s Democratic Republic, Myanmar, Thailand, Vietnam, and Yunnan Province of China. The search strategy was adapted for each database according to its indexing structure and search syntax. In PubMed, both free-text keywords and controlled vocabulary terms (MeSH) were used where appropriate. For Scopus, database-specific search fields and indexing structures were used. The full search strategies for each database are provided in the Supplementary Material S1.

### Study selection

The database search was conducted on 15 March 2026, and all records available up to this date were considered for inclusion. All records retrieved from the database searches were exported to EndNote for reference management and duplicate removal. After deduplication, the remaining records were imported into Rayyan [37] for screening. Study selection was conducted in two stages. First, titles and abstracts of all retrieved records were screened by one reviewer (PLA). To improve the consistency and reliability of study selection, all records with uncertain eligibility, together with a random sample of screened records, were independently reviewed by a second reviewer (AT). Any discrepancies were resolved through discussion. Second, the full texts of potentially relevant studies were independently assessed for inclusion by two reviewers (PLA and AT) according to the predefined eligibility criteria. Any uncertainties regarding study eligibility were resolved through discussion. Studies that did not meet the eligibility criteria during the full-text review were excluded, and reasons for exclusion were recorded. The study selection process was summarized using a PRISMA-ScR flow diagram [33, 34].

### Data charting

Data from included studies were extracted using a standardized data charting form developed for this review (Supplementary Material S2). One reviewer (PLA) extracted data from all included studies. To ensure accuracy and consistency, a subset of studies was independently checked by a second reviewer (AT), and any discrepancies were resolved through discussion. Extracted information included study characteristics such as first author, year of publication, country and study setting, study design, population type, and sample size. Additional data relevant to the review objectives were extracted, including information on malaria epidemiology (e.g., reported species and measures of malaria burden), barriers to accessing malaria prevention, diagnosis, and treatment services, intervention or service delivery approaches targeting migrant populations, and strategies relevant to malaria elimination. The extracted data were organized to facilitate comparison across studies and thematic synthesis.

### Data synthesis

The extracted data were synthesized using descriptive summaries and thematic synthesis. Study characteristics were first summarized descriptively according to publication year, country, study design, and population. Findings were then organized into six thematic domains aligned with the objectives of the review, followed by a narrative synthesis to integrate evidence across studies. A narrative synthesis was conducted to integrate findings across studies, supported by a summary table.

Based on this thematic synthesis, a conceptual framework was developed to illustrate the relationships among the key determinants of malaria transmission among migrant populations in the GMS. The framework was developed inductively by synthesizing recurring themes identified across the included studies and examining the relationships among epidemiological, behavioral, mobility-related, and health system factors. It was informed by patterns observed across the collective body of evidence rather than any individual study and refined through iterative discussion among the authors until consensus was reached.

## Results

### Study selection

**Fig. 1:**
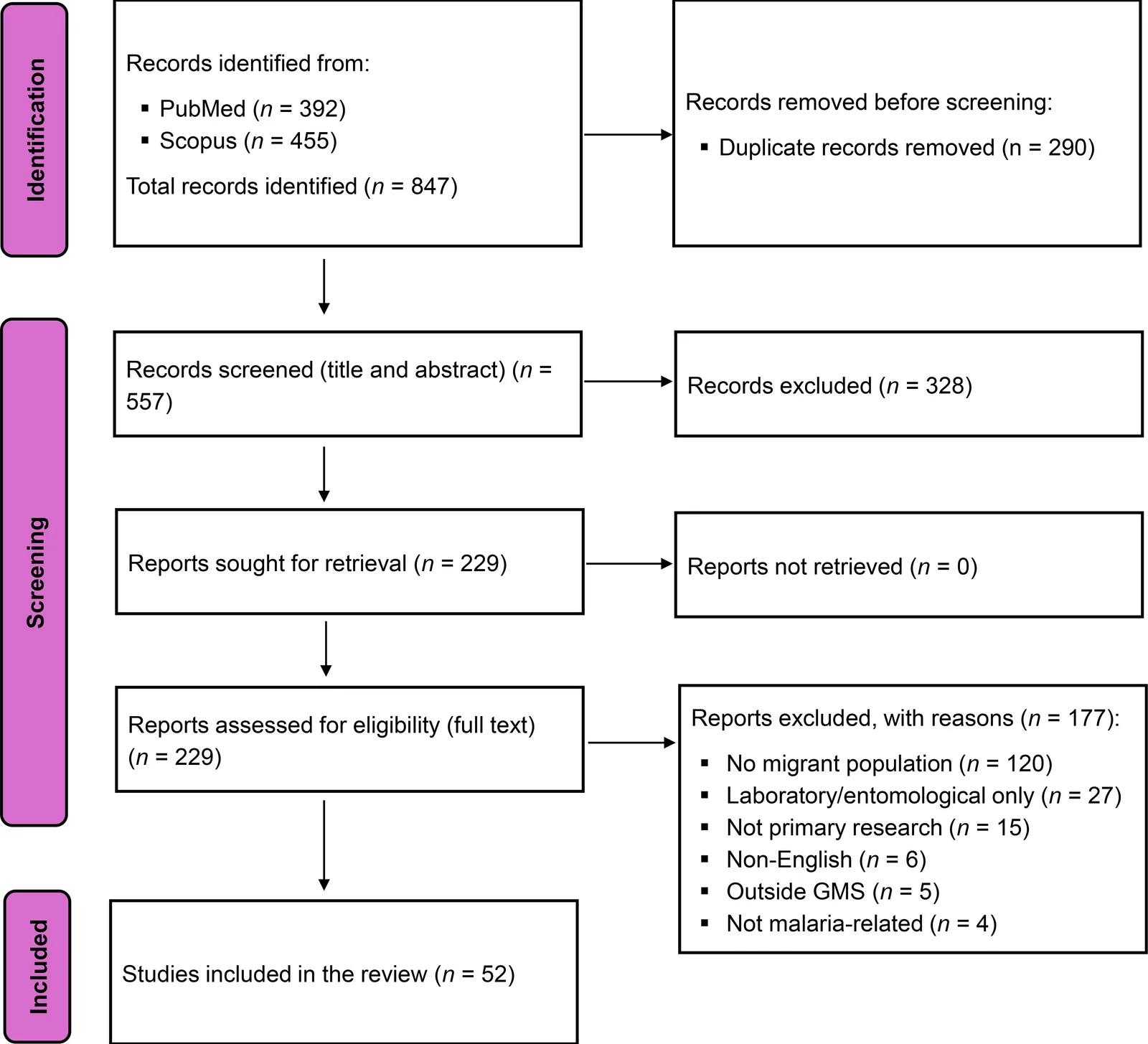
PRISMA flow diagram of study selection

A total of 847 records were identified through database searches (PubMed, *n* = 392; Scopus, *n* = 455). After removal of 290 duplicate records, 557 articles were screened by title and abstract, of which 328 were excluded. A total of 229 full-text articles were assessed for eligibility, and 177 were excluded for reasons including non-migrant populations (*n* = 120), laboratory or entomological studies only (*n* = 27), non-primary research (*n* = 15), non-English language (*n* = 6), studies conducted outside the GMS (*n* = 5), and not malaria-related (*n* = 4). Ultimately, 52 studies were included in this scoping review (Fig. 1).

### Characteristics of included studies

The 52 included studies were conducted across countries of the GMS, with the majority undertaken in Thailand (*n* = 29, 56%), followed by Myanmar (*n* = 15, 29%), Cambodia (*n* = 4), Vietnam (*n* = 2), Lao People’s Democratic Republic (*n* = 1), and the China–Vietnam border region (*n* = 1) (Table 2). Most studies were conducted in malaria-endemic border settings characterized by high population mobility, including forested areas, plantations, agricultural communities, and cross-border transit zones. Most studies employed observational designs, predominantly cross-sectional studies (*n* = 28, 54%), complemented by mixed-methods approaches (*n* = 10, 19%). The remaining studies included retrospective and longitudinal observational studies, randomized intervention trials, case–control studies, implementation studies, therapeutic efficacy studies, and molecular surveillance studies.

The included studies varied considerably in their objectives, methodologies, study populations, and sample sizes, reflecting the diverse epidemiological and operational contexts of malaria among migrant and mobile populations across the GMS. Study populations primarily comprised cross-border migrant workers and other mobile populations engaged in forest-related, agricultural, plantation, and mining occupations (*n* = 49, 94%). A small number of studies focused on specific high-risk groups, including pregnant women [38] and occupational subgroups such as plantation and gold-mining workers [39, 40]. Sample sizes ranged from small qualitative studies to large programmatic datasets involving tens of thousands of participants. Collectively, the included studies spanned more than three decades, reflecting temporal changes in malaria epidemiology, intervention strategies, and surveillance approaches across the region.

Thematic synthesis identified six interrelated domains describing the epidemiological, behavioral, occupational, and health-system factors influencing malaria vulnerability among migrant and mobile populations in the GMS: malaria epidemiology and burden; access to healthcare and treatment-seeking; knowledge, perceptions, and behaviors; prevention practices and vector control; mobility, occupation, and risk factors; and interventions, surveillance, and health system challenges. These domains provided the framework for synthesizing the evidence presented below.

### Thematic synthesis of findings

#### Mobility, occupation, and risk factors

Population mobility emerged as one of the most consistent determinants of malaria vulnerability among migrant and mobile populations across the GMS. Across the included studies (*n* = 8, 15%), mobility was commonly described in terms of cross-border movement, seasonal migration, temporary employment, and work-related mobility rather than directly quantified movement patterns [19, 25, 28, 41–55]. Frequent cross-border movement, seasonal migration, and occupation involving forest work, agriculture, plantations, mining, and other outdoor activities were consistently associated with increased exposure to malaria vectors and elevated infection risk [25, 41–43, 45, 47, 49–53, 56]. Highly mobile populations were less likely to be reached by routine surveillance, preventive interventions, timely diagnosis, and follow-up services, thereby contributing to persistent transmission and posing ongoing challenges for malaria elimination [44, 47, 51, 54, 55, 57]. In addition, social networks, migration pathways, and work-related movement patterns influenced both exposure risk and healthcare access [47, 51, 54, 55]. Collectively, the included studies consistently identified mobility and occupation as important factors influencing malaria exposure, healthcare access, and the effectiveness of malaria control efforts among migrant and mobile populations in the GMS.

#### Malaria epidemiology and burden

Studies (*n* = 13, 25%) examining malaria epidemiology consistently demonstrated that malaria burden among migrant and mobile populations was heterogeneous across the GMS, with transmission concentrated in border regions, forested areas, plantations, and other occupational settings associated with continued malaria exposure [17, 38, 51, 54, 57–65]. Although reported malaria prevalence was generally low in several elimination settings, localized transmission foci persisted in high-risk border areas. Across multiple studies, asymptomatic and submicroscopic infections were consistently identified as important challenges for malaria elimination and were detectable primarily through molecular diagnostic methods [17, 38, 57–59, 63, 65]. These infections were frequently missed by routine microscopy because parasite densities often remained below the detection threshold of conventional diagnostic methods. Consequently, routine surveillance may underestimate the true malaria burden among migrant populations, particularly in low-transmission and elimination settings. Several studies further highlighted that asymptomatic carriers, especially those infected with *Plasmodium vivax*, may constitute an important reservoir capable of sustaining residual malaria transmission [38, 57–59, 63, 65]. While *P. falciparum* remained present in several settings, multiple studies reported an increasing predominance of *P. vivax*, reflecting changing malaria epidemiology across the GMS [59, 63–65]. Two studies also reported resistance-related findings, including detection of artemisinin resistance markers and continued monitoring for potential chloroquine resistance in border settings [40, 66].

#### Access to healthcare and treatment-seeking behaviors

Barriers to timely diagnosis and treatment were consistently reported across studies (*n* = 14, 27%) examining healthcare access among migrant and mobile populations [17, 18, 28, 47, 48, 52, 55, 67–73]. Although many migrants expressed a preference for seeking care through public health services, structural, administrative, and socioeconomic barriers frequently delayed access to timely diagnosis and treatment. Common structural barriers included long travel distance, limited transportation, remote worksites, and indirect financial costs such as travel expenses and loss of income, even where malaria diagnosis and treatment were provided free of charge [17, 18, 28, 55, 68, 70, 72]. Legal and administrative barriers, including lack of documentation and concerns related to migration status, further limited access to formal healthcare services among some migrant populations [18]. Consequently, migrants often followed complex treatment pathways involving self-medication, informal healthcare providers, cross-border healthcare seeking, or delayed presentation to public health facilities [28, 55, 68–70, 72, 73]. Delays in care-seeking beyond 24–48 h were commonly reported and may contribute to continued malaria transmission [18, 68, 70]. Across the included studies, short-term and highly mobile migrants consistently experienced the greatest barriers to timely diagnosis, treatment, and continuity of care compared with more stable migrant populations [17, 55, 58, 68, 69, 73].

#### Knowledge, perceptions, and health-related behaviors

Knowledge, perceptions, and health-related behaviors were examined across a substantial number of included studies (*n* = 15, 29%) [17, 19, 39, 41, 43, 45, 46, 56, 67–70, 74–76]. Previous malaria experience, health education, longer duration of residence, and higher educational attainment were consistently associated with better malaria knowledge and preventive behaviors [39, 45, 46, 68, 69, 75, 76]. Despite these findings, improved knowledge did not consistently translate into appropriate preventive practices or timely healthcare-seeking. Across multiple studies, a persistent gap between knowledge and behavior was observed, with work-related mobility, competing livelihood demands, occupational exposure, and limited physical or financial access to healthcare frequently constraining the adoption of recommended malaria prevention and treatment practices [17, 19, 39, 45, 56, 67, 69, 70, 74, 76]. Misconceptions regarding malaria transmission and treatment also persisted in some migrant populations, suggesting that health education alone may be insufficient unless accompanied by interventions that address the broader social and structural barriers affecting malaria prevention and healthcare access [41, 43, 45, 46, 56].

#### Prevention practices and vector control

Prevention practices and vector control were among the most frequently investigated themes across the included studies (*n* = 19, 37%) [19, 20, 25, 28, 39, 41, 45–48, 50, 56, 63, 64, 69, 72, 74, 76–78]. Although ownership of ITNs or long-lasting insecticidal nets (LLINs) was generally high in many migrant populations, consistent use remained substantially lower across diverse settings. Occupational mobility, forest-related work, outdoor sleeping, irregular sleeping arrangements, and other work-related activities during peak mosquito biting hours were repeatedly identified as major barriers to effective use of conventional vector control measures [19, 25, 28, 39, 46–48, 50, 56, 63, 64, 69, 76, 78]. Programmatic challenges, including insufficient net distribution, inadequate access to replacement nets, and user preferences, also contributed to inconsistent utilization [20, 39, 76–78]. Several studies (*n* = 4) evaluated alternative or complementary vector control approaches tailored to mobile and migrant populations, including insecticide-treated clothing, targeted LLIN distribution strategies, and innovative net delivery programs [20, 74, 77, 79]. These interventions were generally considered feasible and acceptable in specific settings; however, their uptake, adherence, and operational effectiveness varied according to occupational activities, mobility patterns, and local implementation contexts.

#### Interventions, surveillance, and health system challenges

Studies (*n* = 15, 29%) evaluating health system interventions described a range of strategies designed to improve malaria prevention, diagnosis, treatment, and surveillance among migrant and mobile populations [20, 40, 44, 57, 65–68, 77–83]. These included community-based diagnosis and treatment, village health volunteers, mobile health services, digital reporting systems, molecular surveillance, and targeted distribution of prevention tools. Collectively, these approaches aimed to extend service coverage to hard-to-reach populations, strengthen case detection, and improve treatment delivery in highly mobile settings. Despite these efforts, persistent health system challenges were consistently reported. Routine surveillance frequently underrepresented malaria burden among migrant populations because of limited-service coverage, difficulties tracking highly mobile individuals, and reliance on passive case detection [40, 44, 57, 65, 80]. Fragmentation of health services and limited integration of migrant populations into national health systems further constrained equitable access to malaria prevention and treatment [18, 55, 68]. Cross-border population movement further complicated malaria control by disrupting surveillance, continuity of care, and patient follow-up across national boundaries. Several studies described collaborative surveillance and referral mechanisms between neighboring countries; however, these initiatives were geographically limited and not consistently evaluated [40, 44, 80, 82, 83]. Overall, the evidence indicates that maintaining effective malaria control among migrant and mobile populations requires surveillance and service delivery approaches that are responsive to population mobility and cross-border movement.

**Table 2:** Characteristics of studies included in the scoping review (*n* = 52)

ID	Author	Country	Study setting	Study design	Population	Sample size (*n*)	Relevant thematic domain (s)	Key findings
1	Ansari et al. (2025) [67]	Thailand	Thai–Myanmar border	Mixed-methods study	Cross-border migrants	*n* = 729 (quantitative); *n* = 16 (qualitative)	Access to healthcare and treatment seeking; Knowledge, perceptions, and behaviors	45% did not complete primaquine; work demands, mobility, access to care, and perceived risks shaped treatment adherence.
2	Aung et al. (2025) [17]	Thailand	Tak Province (border)	Cross-sectional study	Short-term migrants	*n* = 300	Access to healthcare and treatment seeking; Knowledge, perceptions, and behaviors; Malaria epidemiology and burden	Only 35% sought care within 24 h; care-seeking was influenced by migration, ethnicity, prior malaria, and knowledge; asymptomatic *P. vivax* infections were detected.
3	Aung et al. (2022) [28]	Myanmar	Shan State (border)	Mixed-methods study	Mobile/migrant populations	*n* = 43 (quantitative); *n* = 14 (qualitative)	Prevention practices and vector control; Access to healthcare and treatment-seeking; Mobility, occupation, and risk factors	Worksite migrants had lower ITN/LLIN use than villagers; self-medication was common, and remote worksites, insecurity, and logistics limited access to care.
4	Butraporn et al. (1995) [41]	Thailand	Eastern border provinces	Cross-sectional survey	Mobile populations	*n* = 729	Knowledge, perceptions, and behaviors; Prevention practices and vector control; Mobility, occupation, and risk factors	Knowledge of malaria prevention was limited; mobile villagers frequently moved to forested areas, and self-prevention practices were suboptimal.
5	Canavati et al. (2021) [25]	Vietnam	Forest/farm settings	Cross-sectional study	Mobile/migrant populations	*n* = 1,074	Prevention practices and vector control; Mobility, occupation, and risk factors	Net ownership was high among forest/farm sleepers, but use remained inconsistent; hammock-net preferences and mobility-related sleeping patterns influenced net use.
6	Carrara et al. (2013) [83]	Thailand	Thai–Myanmar border	Observational study	Mobile/migrant populations	*n* ≈ 69,000	Malaria epidemiology and burden; Interventions, surveillance, and health system challenges	Expansion of early diagnosis and treatment (EDT) with ACT was associated with a marked decline in malaria burden among mobile and migrant populations despite the emergence of artemisinin resistance.
7	Carrara et al. (2006) [82]	Thailand	Tak Province	Community-based interventional study	Mobile/migrant populations	population ≈ 450,000	Interventions, surveillance, and health system challenges; Malaria epidemiology and burden	Deployment of community-based early diagnosis using village volunteers and mefloquine–artesunate treatment substantially reduced falciparum malaria cases, hospitalizations, and malaria-related deaths.
8	Chaveepojnkamjorn et al. (2004) [42]	Thailand	Border areas	Population-based case-control study	Cross-border migrants	*n* = 434	Mobility, occupation, and risk factors	Residence in forested areas, prolonged outdoor stay, and frequent migration were independently associated with malaria infection among migrant populations.
9	Chaveepojnkamjorn et al. (2005) [43]	Thailand	Chiang Rai	Cross-sectional study	Cross-border migrants	*n* = 421	Knowledge, perceptions, and behaviors; Mobility, occupation, and risk factors	Poor malaria prevention knowledge, forest exposure, and inadequate preventive practices were associated with malaria infection among migrant populations.
10	Crawshaw et al. (2017) [74]	Myanmar	Plantations	Cluster-randomized non-inferioritycrossover mixed-methods trial	Migrant workers	*n* = 234	Prevention practices and vector control; Knowledge, perceptions, and behaviors	Insecticide-treated clothing was highly acceptable among migrant rubber tappers and may provide a feasible complementary strategy for malaria prevention in outdoor transmission settings.
11	Edwards et al. (2015) [44]	Cambodia	Border crossings	Cross-sectional border surveillance study	Cross-border migrants	*n* = 3,206	Mobility, occupation, and risk factors; Interventions, surveillance and health system challenges	Cross-border screening identified a high burden of asymptomatic and artemisinin-resistant malaria among border-crossing populations, highlighting the need for targeted surveillance and customized interventions at border crossings.
12	Gilder et al. (2025) [38]	Thailand	Border clinics	Case-cohort study	Pregnant migrants	*n* = 4,352	Malaria epidemiology and burden	Submicroscopic malaria during pregnancy was associated with subsequent malaria, maternal anemia, and reduced birth weight, demonstrating that low-density infections contribute substantially to malaria burden in border populations.
13	Hein et al. (2018) [68]	Myanmar	RAI areas	Explanatory mixed-methods study	Migrants	*n* = 3,230	Knowledge, perceptions, and behaviors; Interventions, surveillance, and health system challenges	Despite appropriate care-seeking, timely malaria testing among migrants remained low because of limited awareness of volunteer services and gaps in provider testing practices, indicating important health-system barriers.
14	Hein & Kanchanakhan (2018) [75]	Myanmar	Tanintharyi	Cross-sectional study	Migrants	*n* = 316	Knowledge perceptions, and behaviors	Malaria prevention practices among internal migrants were strongly associated with knowledge, attitudes, education, healthcare accessibility, and exposure to health education.
15	Hlaing et al. (2015) [45]	Myanmar	MARC zones	Cross-sectional study	Migrant workers	*n* = 1,899	Mobility, occupation, and risk factors; Knowledge, perceptions, and behaviors	Highly mobile and less stable migrant workers had poorer malaria knowledge, lower use of LLINs, reduced access to malaria services, and greater vulnerability to malaria, highlighting the need for mobility-adapted malaria control strategies.
16	Kamol-Ratanakul & Prasittisuk (1992) [79]	Thailand	Chonburi	Randomized double-blind field trial	Migrant workers	*n* = 261	Prevention practices and vector control; Interventions, surveillance, and health system challenges	Permethrin-impregnated bed nets significantly reduced malaria episodes among migrant workers and demonstrated an effective complementary vector control strategy in highly endemic settings.
17	Kamolratanakul et al. (1989) [81]	Thailand	Chonburi	Randomized double-blind field trial	Migrants	*n* = 193	Interventions, surveillance, and health system challenges	Weekly chemoprophylaxis with mefloquine plus sulfadoxine-pyrimethamine effectively suppressed malaria among non-immune migrant workers, although concerns regarding drug safety limited its wider applicability.
18	Kitvatanachai et al. (2003) [58]	Thailand	Thai–Cambodia border	Cross-sectional survey	Migrant workers	*n* = 666	Malaria epidemiology and burden; Mobility, occupation, and risk factors	Malaria prevalence among asymptomatic mobile Cambodians was low but frequent cross-border movement, male sex, and lower education were associated with infection, supporting targeted surveillance of mobile populations.
19	Kitvatanachai et al. (2012) [59]	Thailand	Kanchanaburi	Cross-sectional study	Migrants	*n* = 671 (quantitative); *n* = 38 (qualitative)	Malaria epidemiology and burden; Mobility, occupation, and risk factors	Asymptomatic malaria among Myanmar migrant workers was predominantly *P. vivax*, whereas symptomatic infections were mainly *P. falciparum*, highlighting the need for continued surveillance in migrant populations.
20	Krairittichai et al. (2012) [60]	Thailand	Bangkok	Retrospective study	Migrants	*n* = 102,090	Malaria epidemiology and burden	Among over 100,000 immigrant workers undergoing health screening, malaria prevalence was low compared with other infectious diseases, although migrant workers remained an important population for communicable disease surveillance.
21	Kritsiriwuthinan et al. (2011) [57]	Thailand	Bangkok/Samut Sakhon	Cross-sectional survey	Migrants	*n* = 294	Malaria epidemiology and burden; Mobility, occupation, and risk factors; Interventions, surveillance, and health system challenges	Asymptomatic malaria prevalence among foreign migrant workers was low but infections occurred exclusively among Myanmar workers, supporting active surveillance using more sensitive diagnostic methods in migrant populations.
22	Lek et al. (2018) [77]	Cambodia	Border provinces	Cross-sectional survey	Migrant workers	*n* = 702	Prevention practices and vector control; Interventions, surveillance, and health system challenges	The LLIN lending scheme substantially improved mosquito net access among mobile and migrant populations, although gaps remained in knowledge and universal coverage.
23	Linn et al. (2019) [20]	Myanmar	RAI areas	Sequential mixed-methods study	Migrants	*n* = 3,230	Prevention practices and vector control; Interventions, surveillance, and health system challenges	Ownership and utilization of insecticide-treated nets among migrants remained suboptimal because of inadequate net distribution, incomplete migrant mapping, logistical constraints, and user preferences, indicating the need for strengthened distribution strategies.
24	Ly et al. (2017) [69]	Cambodia	Western Cambodia	Respondent-driven cross-sectional survey	Migrants	*n* = 1,501	Knowledge, attitudes, and practices (KAP); Treatment-seeking and healthcare access; Prevention practices and vector control	Although malaria knowledge was generally high, important gaps remained in appropriate treatment-seeking, use of recommended antimalarial therapy, and ownership and utilization of insecticide-treated nets among mobile and migrant populations.
25	Maung et al. (2018) [78]	Myanmar	RAI areas	Cross-sectional study (secondary analysis of community survey)	Migrants	*n* = 3,923	Prevention practices and vector control; Interventions, surveillance, and health system challenges	Despite widespread ownership of bed nets, access to sufficient ITNs/LLINs and their utilization remained inadequate among migrant households, highlighting the need for improved distribution strategies targeting high-risk populations.
26	Meankaew et al. (2010) [80]	Thailand	Border areas	Implementation/ proof-of-concept study	Migrants	program data	Interventions, surveillance, and health system challenges	A mobile phone–based disease and treatment monitoring system substantially improved malaria case follow-up and treatment monitoring among Thai and migrant patients, demonstrating the feasibility of mobile health technologies for malaria surveillance.
27	Muhamad et al. (2011) [66]	Thailand	Border clinic	Prospective therapeutic efficacy study	Migrants	*n* = 130	Interventions, surveillance, and health system challenges	Chloroquine plus primaquine remained highly effective against *P. vivax* along the Thai–Myanmar border, although continued surveillance for emerging chloroquine resistance was recommended.
28	Naing et al. (2020) [70]	Thailand	Southern Thailand	Cross-sectional survey	Migrants	*n* = 414	Treatment-seeking and healthcare access; Knowledge, attitudes, and practices (KAP)	Only about half of migrants with recent fever sought healthcare, self-medication was common, and knowledge that malaria requires treatment was strongly associated with healthcare-seeking behavior.
29	Naing et al. (2021) [46]	Thailand	Southern Thailand	Cross-sectional survey	Migrants	*n* = 414	Knowledge, attitudes, and practices (KAP); Prevention practices and vector control	Malaria knowledge, ITN ownership, and consistent ITN use among migrants remained inadequate, indicating the need for strengthened behavioral change communication and improved ITN coverage.
30	Ngo et al. (2021) [56]	Vietnam	Forest areas	Cross-sectional malaria indicator survey	Mobile populations	*n* = 4,211	Knowledge, attitudes, and practices (KAP); Prevention practices and vector control; Mobility, occupation, and risk factors	Forest-going mobile populations had poor malaria prevention practices, low treated-net use, and frequent self-treatment, highlighting the need for location-specific interventions and strengthened routine surveillance in high-risk transmission settings.
31	Nyunt et al. (2014) [39]	Myanmar	Plantation sites	Mixed-methods cross-sectional study	Migrant workers	*n* = 345	Knowledge, attitudes, and practices (KAP); Prevention practices and vector control	Although ITN ownership among migrant plantation workers was high, consistent utilization remained suboptimal because of behavioral, occupational, and practical barriers, highlighting the need for behavior change interventions and continuous LLIN distribution.
32	Nyunt et al. (2021) [19]	Myanmar	Mining/forest sites	Mixed-methods cross-sectional study	Migrants	*n* = 102	Knowledge, attitudes, and practices (KAP); Prevention practices and vector control; Mobility, occupation, and risk factors	Residual malaria among migrant workers persisted because of low malaria knowledge, inconsistent LLIN use, and occupational exposure, indicating that migrant-specific prevention and health promotion strategies are needed.
33	Nyunt et al. (2017) [40]	Myanmar	MARC zones	Cross-sectional molecular surveillance study	Migrant workers	*n* = 100	Interventions, surveillance, and health system challenges	Artemisinin resistance markers, including K13 mutations, were common among migrant goldmine workers, supporting strengthened molecular surveillance and containment efforts in Myanmar.
34	Peeters Grietens et al. (2015) [47]	Cambodia	Ratanakiri	Mixed-methods study	Migrants	n ≈ large	Mobility occupation, and risk factors; Prevention practices and vector control; Treatment-seeking and healthcare	Different forms of human mobility created distinct malaria risks, demonstrating that indigenous forest-goers and migrant workers require tailored malaria prevention, surveillance, and healthcare strategies rather than uniform interventions.
35	Than et al. (2017) [76]	Myanmar	Bago Region	Cross-sectional study (secondary analysis)	Migrants	*n* = 2,484	Knowledge, attitudes, and practices (KAP); Prevention practices and vector control	Although most migrant households owned at least one bed net, adequate bed-net coverage, knowledge, and utilization remained insufficient, particularly among seasonal migrants, highlighting the need for improved distribution and education strategies.
36	Pongvongsa et al. (2019) [48]	Lao PDR	Savannakhet	Cross-sectional study	Migrants	*n* = 391	Treatment-seeking and healthcare access; Prevention practices and vector control; Mobility, occupation, and risk factors	No malaria infections were detected among registered foreign migrant workers, although forest-related activities and suboptimal use of insecticide-treated nets suggested continued malaria vulnerability and the need for targeted health education.
37	Rakprasit et al. (2017) [71]	Thailand	Border/industrial areas	Retrospective analysis of national surveillance data	Migrants	n = large	Epidemiology; Treatment-seeking and healthcare access	Migrant workers had substantially higher healthcare utilization for malaria, tuberculosis, and sexually transmitted infections than Thai workers, particularly among agricultural workers, highlighting important occupational health disparities.
38	Rattanapunya et al. (2015) [61]	Thailand	Mae Sot	Retrospective study	Migrants	*n* = 256	Epidemiology	Malaria–HIV co-infection was uncommon but increased over time among malaria patients attending a migrant clinic, indicating the need for continued surveillance.
39	Rattanapunya et al. (2015) [62]	Thailand	Border clinic	Retrospective study	Migrants	*n* = 867	Epidemiology	HIV co-infection was associated with higher parasite density and lower hemoglobin levels among malaria patients, suggesting increased malaria severity despite a low prevalence of co-infection.
40	Singhanetra-Renard (1993) [49]	Thailand	Northern Thailand	Mixed-methods study	Mobile populations	*n* = 50	Mobility, occupation, and risk factors	Agricultural expansion, forest-related livelihoods, and cross-border mobility increased human-vector contact and malaria transmission, demonstrating the need for mobility-informed malaria control strategies.
41	Soe et al. (2017) [50]	Myanmar	Plantation regions	Cross-sectional analytic study	Migrants	*n* = 406	Mobility, occupation, and risk factors; Prevention practices and vector control	Malaria among internal migrants was associated with male sex, early-morning outdoor activity, inconsistent or poor-quality bed net use, alcohol consumption, and lack of malaria health education, highlighting the importance of targeted behavioral interventions.
42	Sripoorote et al. (2024) [18]	Thailand	Border areas	Cross-sectional study	Migrants	*n* = 300	Treatment-seeking and healthcare access	Only about one-quarter of Myanmar migrants had adequate access to comprehensive malaria services. Older age, low income, Karen ethnicity, poor perceptions, and poor preventive and health-seeking practices were significant barriers to malaria service access.
43	Sriwichai et al. (2017) [51]	Thailand	Border village	Retrospective surveillance and entomological study	Migrants	*n* = 4,425 visits	Epidemiology, Mobility, occupation, and risk factors	Recent migrants were significantly more likely to acquire imported *P. falciparum* infections, whereas *P. vivax* transmission was closely associated with local vector abundance, demonstrating distinct cross-border transmission dynamics.
44	Thu & Pumpaibool (2018) [52]	Myanmar	Border region	Unmatched case-control study	Migrants	*n* = 320	Mobility, occupation, and risk factors; Treatment-seeking and healthcare access	Inter-regional, seasonal, and non-contract migration increased malaria risk, while poor protective behaviors and limited access to malaria services were the strongest independent predictors of malaria infection.
45	Tipmontree et al. (2009) [53]	Thailand	Kanchanaburi	Cross-sectional study	Migrants	*n* = 192	Mobility, occupation, and risk factors	Forest-related occupations were the strongest predictor of self-reported malaria among migrants, emphasizing that occupational exposure rather than knowledge alone drives malaria risk in border populations.
46	Tu et al. (2022) [63]	China	China–Vietnam border	Cross-sectional survey	Migrants	*n* = 108	Prevention practices and vector control; Epidemiology	Vietnamese migrants at the China–Vietnam border had poor malaria knowledge and inconsistent use of preventive measures despite high bed-net ownership. PCR detected asymptomatic *P. falciparum* and *P. vivax* infections, supporting proactive surveillance and targeted health education among cross-border migrants.
47	Wai et al. (2014) [72]	Myanmar	Border regions	Mixed-methods cross-sectional study	Migrants	*n* = 408	Treatment-seeking and healthcare access; Prevention practices and vector control	Scattered settlements, irregular work schedules, and transportation barriers delayed access to malaria diagnosis and treatment among migrant workers. Knowledge of appropriate treatment and drug resistance was limited, highlighting the need for community-based malaria services and improved access to LLINs and early diagnosis.
48	Wangroongsarb et al. (2016) [54]	Thailand	Ranong	Respondent-driven sampling with PCR and serology	Migrants	*n* = 1,242	Mobility, occupation, and risk factors; Epidemiology	Malaria prevalence among Myanmar migrants in Ranong was very low despite ongoing population movement. Occupational and travel networks suggested a currently limited risk of spreading artemisinin-resistant parasites, although continued surveillance of migrant populations.
49	Wangroongsarb et al. (2011) [73]	Thailand	Thai–Cambodia border	Respondent-driven sampling survey	Migrants	*n* = 1,719	Treatment-seeking and healthcare access	Long-term Myanmar migrants generally had good access to Thai healthcare, whereas highly mobile Cambodian migrants had poor access to health information and malaria services and often returned to Cambodia or self-treated when ill, indicating substantial gaps in cross-border malaria care.
50	Wangroongsarb et al. (2012) [64]	Thailand	Eastern border	Cross-sectional employer-based study	Migrants	*n* = 5,371	Epidemiology; Prevention practices and vector control	Malaria prevalence among migrants along the Thai–Cambodian border was very low, with only *P. vivax* detected. Cambodian seasonal workers had limited Thai language proficiency and lower bed-net ownership, emphasizing the need for language-appropriate health education and preventive interventions.
51	Win et al. (2017) [55]	Myanmar	Border regions	Mixed-methods study (secondary quantitative analysis + FGDs + IDIs)	Migrant workers	*n* = 9,603	Treatment-seeking and healthcare access; Mobility, occupation, and risk factors	Highly mobile migrant workers in unstable worksites rarely sought malaria care from the public sector. Preference for informal providers and self-medication was driven by convenience, transportation costs, work constraints, trust, and poor access to rural health services. Longer duration of stay, malaria knowledge, and exposure to health education increased public-sector treatment-seeking. The study recommends innovative outreach services tailored to mobile workers.
52	Kritsiriwuthinan & Ngrenngarmlert (2011) [65]	Thailand	Multiple provinces	Cross-sectional molecular surveillance	Migrants	*n* = 241	Epidemiology; Surveillance and diagnosis	Molecular screening (semi-nested multiplex PCR) detected substantially more asymptomatic malaria infections than microscopy among migrant workers, with *P. falciparum* predominating. The findings support incorporating molecular malaria screening into migrant health surveillance to identify low-density infections that conventional microscopy may miss.

[Abbreviations: ITN, insecticide-treated net; LLIN, long-lasting insecticidal net; RDT, rapid diagnostic test; ACT, artemisinin-based combination therapy; PCR, polymerase chain reaction.]

### Conceptual framework of malaria transmission among migrant and mobile populations

**Fig. 2:**
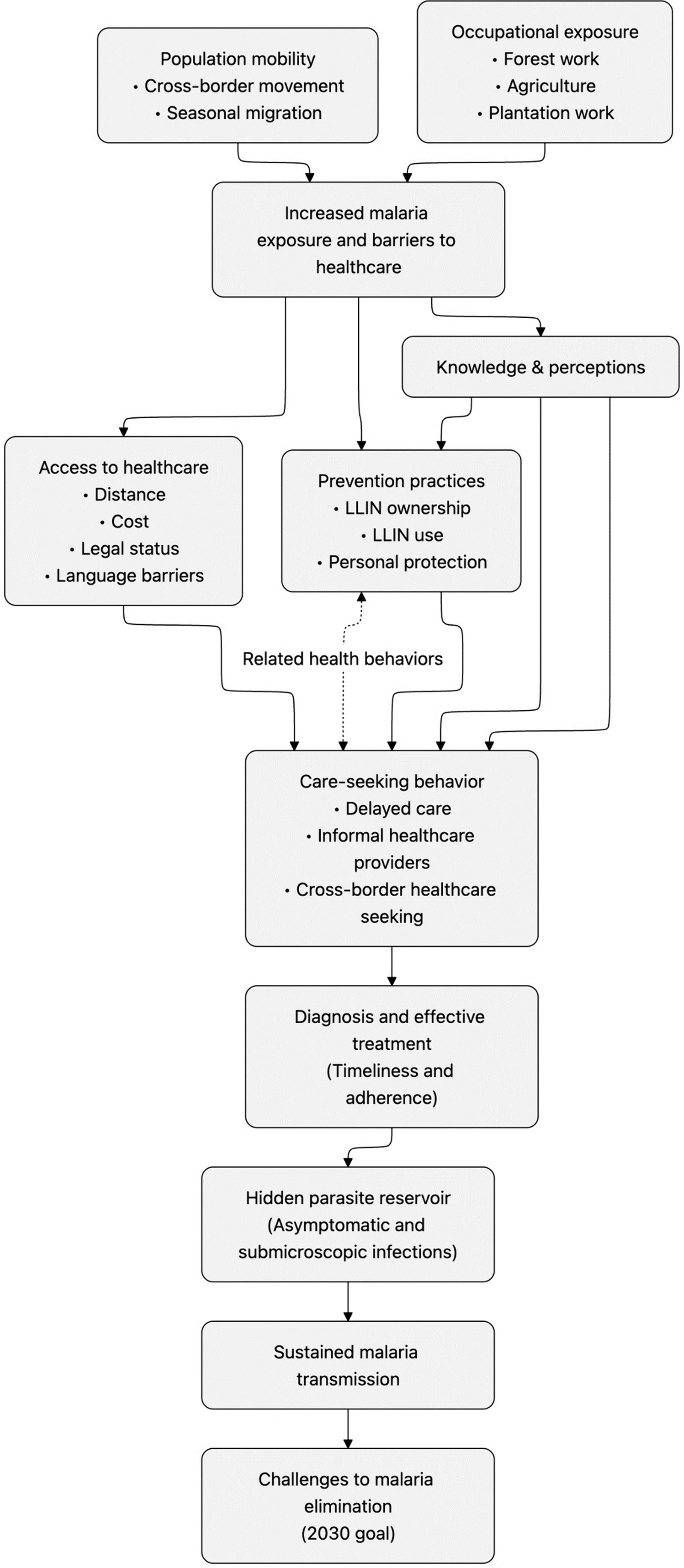
Conceptual framework illustrating the relationships between population mobility, occupational exposure, healthcare access, prevention practices, care-seeking behavior, and malaria transmission among migrant and mobile populations in the GMS

Based on the thematic synthesis of the included studies, a conceptual framework was developed to integrate the major themes emerging from the review and illustrate the conceptual relationships among factors influencing malaria transmission among migrant and mobile populations in the GMS (Fig. 2). The framework represents relationships synthesized across the included studies rather than direct causal pathways. To enhance clarity, it focuses on the principal conceptual pathways linking upstream determinants to sustained malaria transmission rather than depicting all intervention and health system components identified in the review. The framework illustrates population mobility and occupational exposure as parallel upstream determinants that increase malaria exposure while creating structural barriers to healthcare. These upstream determinants influence three key intermediate domains: access to healthcare, knowledge and perceptions, and prevention practices. Knowledge and perceptions shape both prevention practices and care-seeking behavior, while prevention practices and care-seeking behavior are presented as related health behaviors. Barriers such as distance, cost, legal status, and language differences contribute to delayed care-seeking, diagnosis, and effective treatment, whereas gaps between knowledge and behavior result in suboptimal implementation of preventive measures. Together, these interacting factors contribute to the persistence of a hidden parasite reservoir comprising asymptomatic and submicroscopic infections that frequently remain undetected by routine surveillance. This hidden reservoir sustains malaria transmission and presents an ongoing challenge to achieving malaria elimination among migrant and mobile populations in the region.

## Discussion

This scoping review synthesizes evidence from 52 studies examining malaria among migrant and mobile populations in the GMS. Despite substantial regional progress toward malaria elimination, the findings demonstrate that these populations continue to play a critical role in sustaining residual transmission. Malaria burden among migrants was generally low but highly heterogeneous, with persistent transmission concentrated in border and forested areas. Similar patterns have been observed in other elimination settings, particularly across the Asia-Pacific region and other low-transmission settings, where residual transmission has become increasingly concentrated among mobile, migrant, and other hard-to-reach populations [84–91]. A consistent finding across studies was the presence of asymptomatic and submicroscopic infections, a common feature of malaria epidemiology in endemic settings, but particularly relevant in migrant populations, where mobility and barriers to healthcare access may further limit detection and timely treatment [92–94]. These hidden infections suggest that conventional surveillance systems likely underestimate the true burden of malaria among migrants, particularly in low-transmission settings. Evidence from both within and beyond the GMS has highlighted the importance of these reservoirs in sustaining transmission, in recent years especially for *P. vivax* [94–97]. Beyond epidemiological patterns, this review highlights that structural, behavioral, occupational, and health system factors interact to sustain malaria transmission among mobile and migrant populations. Limited access to timely diagnosis and treatment, suboptimal use of preventive tools, and high levels of mobility collectively contribute to ongoing transmission dynamics. These findings emphasize that achieving malaria elimination requires not only reducing incidence but also addressing the complex vulnerabilities of migrant populations.

While this review identified asymptomatic and submicroscopic malaria as important hidden reservoirs of transmission among migrant and mobile populations, very few studies examined how these concepts were understood or perceived by affected communities. This represents an important evidence gap, as individuals without symptoms may not perceive themselves to be at risk or understand the rationale for malaria testing or treatment in the absence of illness [98, 99]. Experiences from targeted malaria elimination programs have demonstrated that effective community engagement and culturally appropriate communication are essential for explaining the role of asymptomatic infections and fostering trust in interventions aimed at interrupting transmission [100]. Future research should explore community perceptions of asymptomatic malaria and evaluate culturally appropriate approaches to improve understanding and acceptance of elimination-focused interventions among migrant and mobile populations. Improving community understanding of asymptomatic malaria may be particularly important for migrant and mobile populations, where language barriers, cultural diversity, frequent mobility, and limited engagement with formal health systems may further hinder the uptake of elimination-focused interventions. Building trust through meaningful community engagement and culturally appropriate communication will be essential to ensure that malaria elimination strategies are not only scientifically effective but also acceptable, equitable, and sustainable among populations at greatest risk.

The findings of this review highlight four priority areas for malaria elimination strategies in the GMS. First, the high prevalence of asymptomatic and submicroscopic infections indicates that reliance on passive case detection and routine microscopy is insufficient for detecting all cases. Enhanced surveillance approaches, including targeted screening and the use of more sensitive diagnostic tools, are likely necessary to identify hidden infections in high-risk populations [101–105]. Second, persistent barriers to healthcare access highlight the need for more flexible and decentralized service delivery models. Although migrants often prefer public health services, structural constraints, such as mobility, work demands, and legal or financial barriers, frequently delay care-seeking [13, 17, 18, 49, 106–109]. Expanding community-based services, mobile clinics, and cross-border referral mechanisms may improve access to timely diagnosis and treatment for symptomatic individuals. However, these approaches also present an important operational challenge in malaria elimination settings. As transmission declines, malaria programs often scale back community-based services because of reduced case numbers and shifting health priorities. At the same time, the remaining transmission is increasingly concentrated among highly mobile and hard-to-reach populations who may rely most heavily on these services. Sustaining targeted, flexible, and context-specific service delivery models will therefore remain critical during the final stages of malaria elimination. Third, the consistent gap between knowledge and preventive behavior suggests that conventional health education approaches alone are insufficient [110–114]. While ITNs and LLINs remain key preventive tools in the region, their effectiveness is often limited among mobile populations due to occupational exposure, outdoor activities, and irregular sleeping arrangements. Interventions should therefore be adapted to the lived realities of migrant populations, particularly those engaged in forest-related or outdoor occupations. Several studies described complementary strategies, such as insecticide-treated clothing, hammock nets, and targeted distribution approaches tailored to mobile and occupational settings. Although these interventions show promise for addressing the unique exposure patterns of migrant and mobile populations, the available evidence on their effectiveness remains limited. Future studies should evaluate not only the effectiveness of these interventions but also their feasibility, acceptability, cost-effectiveness, and sustainability among diverse migrant and mobile populations. Finally, cross-border mobility remains a central challenge for malaria elimination in the region [1, 10, 115]. Movement between endemic areas can facilitate both parasite transmission and the spread of antimalarial drug resistance [116–119]. Strengthening regional coordination, including harmonized case management guidelines, interoperable surveillance systems, data-sharing mechanisms, and cross-border referral policies, will be essential to address these transnational dynamics effectively. Experiences from other malaria elimination settings in the Asia-Pacific region have similarly highlighted that sustained cross-border collaboration and coordinated surveillance are critical for maintaining progress towards elimination, particularly in areas with frequent population movement.

Importantly, many of the barriers identified in this review are not unique to migrant and mobile populations. Rural and underserved host communities across the GMS also experience challenges in accessing malaria services because of geographic remoteness, limited healthcare infrastructure, workforce shortages, and financial constraints [120]. However, migrant and mobile populations often encounter these existing barriers in combination with additional structural vulnerabilities, including language barriers, legal or documentation status, cross-border mobility, temporary employment, unfamiliarity with local health systems, and reduced entitlement to health and social protection services [1, 11, 13, 18, 26–30, 121]. These intersecting vulnerabilities amplify existing inequities in access to malaria prevention, diagnosis, and treatment, placing migrant and mobile populations at disproportionately greater risk of delayed care, missed infections, and continued malaria transmission. These findings suggest that malaria elimination strategies should move beyond geographically targeted approaches and incorporate equity-oriented interventions that address the additional structural barriers faced by migrant and mobile populations.

The findings of this review, together with evidence from broader malaria elimination literature, indicate that mobile and migrant populations are disproportionately affected by malaria and frequently remain underserved by conventional health systems because of intersecting structural, social, and health system barriers [13, 17, 18, 122]. Studies from multiple regions have demonstrated that mobility, occupational exposure, and structural barriers contribute to persistent transmission, particularly in low-incidence settings approaching elimination [52, 123–138]. The identification of asymptomatic and submicroscopic infections aligns with a growing body of literature emphasizing hidden reservoirs in malaria transmission [93, 139–143]. Similarly, the observed gap between knowledge and practice reflects well-documented limitations of behavior change interventions that do not adequately address structural determinants of health. Beyond confirming previous evidence, this review provides a comprehensive regional synthesis integrating epidemiological, behavioral, occupational, and health system perspectives to better understand how multiple factors collectively shape malaria vulnerability among migrant and mobile populations. This integrated perspective is particularly relevant for informing elimination strategies in settings characterized by high population mobility.

Despite the growing body of evidence, several important knowledge gaps remain. First, there is limited longitudinal and intervention-focused research evaluating the effectiveness of tailored strategies for migrant populations. Most studies were cross-sectional, limiting the ability to assess causal relationships and long-term impacts. Furthermore, the evidence base was uneven across countries, with most studies conducted in Thailand and Myanmar and relatively limited evidence from other GMS settings. This imbalance likely reflects differences in research capacity, accessibility, and programmatic investment across countries, rather than the true distribution of malaria risk among migrant populations. Consequently, the existing literature may overrepresent well-studied settings while underrepresenting regions where transmission and cross-border mobility remain important but are more challenging to investigate. Second, while barriers to healthcare access and prevention are well documented, fewer studies have evaluated scalable and sustainable solutions to address these challenges. Implementation research is needed to identify effective and context-appropriate service delivery models for highly mobile populations. Third, evidence on specific high-risk subgroups remains limited. Only a small number of studies examined groups such as pregnant women and occupational subgroups, including plantation and gold-mining workers, despite their potentially elevated risk of malaria exposure and adverse outcomes [38–40]. In addition, key populations such as short-term migrants, undocumented workers, and forest-goers remain underrepresented, despite their likely contribution to ongoing transmission [13, 17, 52, 144, 145]. More granular research is required to better understand heterogeneity within migrant populations. Finally, although concerns regarding the spread of antimalarial drug resistance were noted, there is limited empirical evidence directly linking migration patterns to resistance dynamics [8, 134, 135, 146–149]. This represents a critical area for future research in the context of regional elimination efforts.

This scoping review provides a comprehensive synthesis of evidence across multiple countries in the GMS, integrating findings from diverse study designs and population groups. The structured approach to data extraction and thematic synthesis enabled a broad understanding of malaria epidemiology, behaviors, and health system challenges among migrant populations. However, several limitations should be considered. First, although PubMed and Scopus provide broad coverage of the peer-reviewed literature, relevant studies indexed exclusively in other databases may not have been identified. The review was also restricted to English-language publications, which may have excluded relevant studies from local sources. Second, as a scoping review, no formal assessment of study quality or risk of bias was conducted. Third, heterogeneity in study design, populations, and outcome measures may affect comparability across studies. However, this heterogeneity reflects the diversity of real-world contexts and allowed for a broader and more comprehensive synthesis of evidence across epidemiological, behavioral, and health system dimensions. In addition, most included studies were conducted in specific border or high-transmission settings and may not be nationally representative, limiting generalizability. Additionally, studies reporting malaria importation, particularly along the China–Myanmar border, were excluded when migrant or mobile populations were not explicitly defined or characterized. While such studies provide important insights into cross-border transmission dynamics, the lack of clear population definitions limited their inclusion, potentially resulting in underrepresentation of evidence related to importation-driven transmission. The included evidence spans multiple decades, during which malaria epidemiology, intervention strategies, and migration patterns have evolved. Finally, this review focused on migrant populations within the GMS and did not fully capture migration dynamics beyond the region or other related health factors, such as co-infections or conditions affecting treatment outcomes, which warrant further investigation.

## Conclusions

Migrant and mobile populations remain a critical challenge to malaria elimination in the GMS. Despite overall declines in malaria burden, residual transmission persists because of a combination of asymptomatic infections, structural barriers to healthcare access, suboptimal preventive practices, and high levels of population mobility. Achieving malaria elimination by 2030 will require moving beyond conventional approaches toward tailored, equitable, context-specific strategies for migrant and mobile populations. Strengthening surveillance systems to detect hidden infections, expanding access to flexible and decentralized healthcare services, adapting vector control interventions to occupational and mobile settings, and enhancing cross-border collaboration will be essential to address the complex dynamics of malaria transmission in the region. A sustained focus on migrant populations, supported by targeted research and coordinated regional action, will be critical to ensuring that malaria elimination efforts in the GMS are both equitable and effective.

## Supplementary Information

Below is the link to the electronic supplementary material.

Supplementary Material 1

Supplementary Material 2

## Data Availability

All data generated or analyzed during this study are included in this published article and its supplementary materials. The full list of included studies and extracted data are available from the corresponding author upon reasonable request.

## Abbreviations

ACT: Artemisinin based combination therapy
GMS: Greater Mekong Subregion
ITN: Insecticide-treated net
LLIN: Long-lasting insecticidal net
PCR: Polymerase chain reaction
RDT: Rapid diagnostic test
