# Supplementary material for: Malaria among migrant populations in the Greater Mekong Subregion: a scoping review of evidence and implications for malaria elimination
Source: Int J Equity Health. 2026 Jul 28;25:256. doi: 10.1186/s12939-026-02953-5 (PMC13700450; doi:10.1186/s12939-026-02953-5)
Supplement: Supplementary file 1 [file 12939_2026_2953_MOESM1_ESM.docx]

**Standardized data charting form used for data extraction.**

| **Variable** | **Description** |
| --- | --- |
| First author | Name of first author |
| Publication year | Year of publication |
| Country | Country where study conducted |
| Study setting | Border, forest, plantation, etc. |
| Study design | Cross-sectional, mixed methods, cohort, etc. |
| Population | Migrants, seasonal workers, forest-goers, etc. |
| Sample size | Number of participants |
| Malaria outcome | Species, prevalence, incidence, etc. |
| Main findings | Principal results |
| Review domain | Epidemiology, healthcare access, prevention, mobility, interventions, etc. |
| Notes | Additional remarks |
