# Supplementary material for: Malaria among migrant populations in the Greater Mekong Subregion: a scoping review of evidence and implications for malaria elimination
Source: Int J Equity Health. 2026 Jul 28;25:256. doi: 10.1186/s12939-026-02953-5 (PMC13700450; doi:10.1186/s12939-026-02953-5)
Supplement: Supplementary file 2 [file 12939_2026_2953_MOESM2_ESM.docx]

**Search String**

| **Pubmed** | | |
| --- | --- | --- |
| **Date** | **Search String** | **No of Hits/Citations** |
| 15/03/2026 | (malaria[MeSH Terms] OR malaria[Title/Abstract] OR plasmodium[Title/Abstract] OR falciparum[Title/Abstract] OR vivax[Title/Abstract]) | 129,812 |
| 15/03/2026 | (malaria[MeSH Terms] OR malaria[Title/Abstract] OR plasmodium[Title/Abstract] OR falciparum[Title/Abstract] OR vivax[Title/Abstract])  AND  ("Emigrants and Immigrants"[MeSH]  OR migrant*[Title/Abstract]  OR migration[Title/Abstract]  OR "migrant worker*"[Title/Abstract]  OR "mobile population*"[Title/Abstract]  OR "cross-border"[Title/Abstract]  OR "border population*"[Title/Abstract]  OR "seasonal worker*"[Title/Abstract]  OR refugee*[Title/Abstract]  OR displaced[Title/Abstract]  OR "internally displaced person*"[Title/Abstract]) | 2,639 |
| 15/03/2026 | (malaria[MeSH Terms] OR malaria[Title/Abstract] OR plasmodium[Title/Abstract] OR falciparum[Title/Abstract] OR vivax[Title/Abstract])  AND  ("Emigrants and Immigrants"[MeSH]  OR migrant*[Title/Abstract]  OR migration[Title/Abstract]  OR "migrant worker*"[Title/Abstract]  OR "mobile population*"[Title/Abstract]  OR "cross-border"[Title/Abstract]  OR "border population*"[Title/Abstract]  OR "seasonal worker*"[Title/Abstract]  OR refugee*[Title/Abstract]  OR displaced[Title/Abstract]  OR "internally displaced person*"[Title/Abstract])  AND  ("Greater Mekong Subregion"[Title/Abstract]  OR Cambodia[Title/Abstract]  OR "Lao PDR"[Title/Abstract]  OR Laos[Title/Abstract]  OR Myanmar[Title/Abstract]  OR Burma[Title/Abstract]  OR Thailand[Title/Abstract]  OR Vietnam[Title/Abstract]  OR Yunnan[Title/Abstract]  OR "Yunnan Province"[Title/Abstract]  OR China[Title/Abstract]) | 392 |

| **Scopus** | | |
| --- | --- | --- |
| **Date** | **Search String** | **No of Hits/Citations** |
| 15/03/2026 | TITLE-ABS-KEY (malaria OR plasmodium OR falciparum OR vivax) | 177,702 |
| 15/03/2026 | TITLE-ABS-KEY (malaria OR plasmodium OR falciparum OR vivax)  AND  TITLE-ABS-KEY (migrant*  OR migration  OR "migrant worker*"  OR "mobile population*"  OR "mobile and migrant population*"  OR "cross-border"  OR "border population*"  OR "border malaria"  OR "seasonal worker*"  OR refugee*  OR displaced  OR "internally displaced person*") | 4,801 |
| 15/03/2026 | TITLE-ABS-KEY (malaria OR plasmodium OR falciparum OR vivax)  AND  TITLE-ABS-KEY (migrant*  OR migration  OR "migrant worker*"  OR "mobile population*"  OR "mobile and migrant population*"  OR "cross-border"  OR "border population*"  OR "border malaria"  OR "seasonal worker*"  OR refugee*  OR displaced  OR "internally displaced person*")  AND  TITLE-ABS-KEY ("Greater Mekong Subregion" OR Cambodia  OR "Lao PDR"  OR Laos  OR Myanmar  OR Burma  OR Thailand  OR Vietnam  OR Yunnan  OR "Yunnan Province") | 455 |
